# Tongue feature-based model for assessing disease activity in patients with rheumatoid arthritis

**DOI:** 10.3389/fphar.2025.1651557

**Published:** 2025-09-23

**Authors:** Yuxin Han, Zihan Wang, Meiqi Lan, Yuting Bian, Guangyao Chen, Jiafeng Ao, Haolu Wu, Weichao Li, Qingwen Tao, Yuan Xu, Jianming Wang

**Affiliations:** ^1^ Graduate School, Beijing University of Chinese Medicine, Beijing, China; ^2^ Department of Traditional Chinese Medicine Rheumatology, China-Japan Friendship Hospital, Beijing, China

**Keywords:** disease activity, rheumatoid arthritis, tongue characteristics, laboratory indexes, clinical predictive model

## Abstract

**Introduction:**

Tongue features, which are emerging imaging-based biomarkers, have been integrated into predictive models for various diseases. However, their role in assessing rheumatoid arthritis (RA) activity remains unexplored. This study aims to develop a clinically applicable model for assessing RA activity by analyzing the relationship between tongue features and laboratory indicators.

**Methods:**

We enrolled 227 patients who visited the Department of Traditional Chinese Medicine Rheumatology, China-Japan Friendship Hospital, from April 2021 to March 2023. Patients were stratified into remission/low-activity (n = 75) and moderate/high activity (n = 152) groups. Multivariable logistic regression was used to develop two predictive models: Model 1 (based on laboratory parameters) and Model 2 (Model 1 plus tongue features). Both models were presented as nomograms and web-based calculators. Model discrimination was evaluated using receiver operating characteristic curves, calibrated via calibration plots, and clinical utility was determined using decision curve analysis.

**Results:**

Multivariable logistic regression identified white blood cell (WBC), hemoglobin (HGB), platelets (PLT), and IgA as predictors in Model 1, while Model 2 incorporated WBC, HGB, greasy coating and sublingual varicosity. Model 2 outperformed Model 1, achieving an area under the curve of 0.846 (95% confidence interval = 0.740–0.951), with a sensitivity of 0.63 and specificity of 0.826. A nomogram and online calculator were developed from this optimized model for clinical use.

**Conclusion:**

We have developed a preliminary RA disease activity assessment model integrating tongue features and laboratory parameters. This model shows high accuracy and considerable potential for clinical utility.

## 1 Introduction

Rheumatoid arthritis (RA) is a systemic autoimmune disease characterized by chronic inflammation of the synovial membranes (Littlejohn and Monrad, 2018). Clinically, it presents with morning stiffness, joint pain, swelling, and progressive joint destruction. In advanced stages, RA may cause systemic complications, including interstitial lung disease and cardiovascular disorders. Global epidemiological data indicate a prevalence of approximately 0.5%–1% (Hassen et al., 2024). In China, the prevalence is 0.42%, with RA-related mortality accounting for 20% of deaths attributed to arthritis and musculoskeletal diseases (Jin et al., 2017). This public health burden underscores the importance of early intervention.

Treat-to-target (T2T) strategies, grounded in evidence-based medicine, are widely accepted in RA management. Their primary goal is to achieve clinical remission or maintain low disease activity through dynamic and continuous monitoring of disease progression. Accurate assessment of disease activity is clinically important for both quantitative prognostic evaluation and for guiding stepwise optimization of therapy (Anderson et al., 2012). Currently, the American College of Rheumatology (ACR) recommends multidimensional composite systems to evaluate RA activity, including the Disease Activity Score using 28-joint counts (DAS28), Simplified Disease Activity Index (SDAI), Clinical Disease Activity Index (CDAI), Patient Activity Scale (PAS), PAS-Ⅱ, and Routine Assessment of Patient Index Data 3 (RAPID-3). These measures incorporate parameters such as tender joint count (TJC), swollen joint count (SJC), and patient global assessment. Although validated and widely used, they require specialized training, particularly for assessing joint tenderness and swelling, limiting their use in non-specialist settings and creating challenges in primary care or patient self-management. Consequently, developing simplified yet reliable disease activity assessment tools are essential. Such methods can broaden use among non-rheumatologist healthcare providers and enable patient self-monitoring, thereby promoting individualized, responsive treatment strategies consistent with the T2T paradigm.

Imaging features are valuable for accurate RA diagnosis and prognosis. They reveal the extent of joint structural damage and, more importantly, enable quantitative assessment of disease progression, providing predictive insights into future clinical outcomes. Quantitative analysis of bone marrow edema via magnetic resonance imaging effectively predicts structural progression in patients in clinical remission (Gandjbakhch et al., 2011; Baker et al., 2014). Similarly, predictive models combining musculoskeletal ultrasound features with clinical risk factors can help identify individuals at high risk for bone erosion (Yan et al., 2025). Notably, tongue features, classified as imaging characteristics, are non-invasive, simple, and cost-effective. Therefore, tongue features are increasingly incorporated into clinical prediction models. Duan et al. developed a coronary artery disease diagnostic model based on tongue features, demonstrating robust performance (Duan et al., 2024). Li et al. report that incorporating tongue features significantly enhanced the accuracy of conventional machine learning models for diabetes risk prediction (Li et al., 2021). Collectively, these findings highlight the complementary diagnostic value of tongue features in systemic disease assessment and suggest that combining them with traditional serum biomarkers could yield more practical, multimodal RA risk assessment models.

Building on this evidence, this study aims to investigate the potential of objective tongue features as a complementary tool for assessing RA disease activity. Using machine learning algorithms, we integrate tongue parameters, blood biochemical indicators, and immune biomarkers to develop a disease activity assessment framework tailored to the Chinese population, thereby offering a potential adjunctive tool for clinical evaluation.

## 2 Materials and methods

### 2.1 Study design and participant selection

**FIGURE 1:**
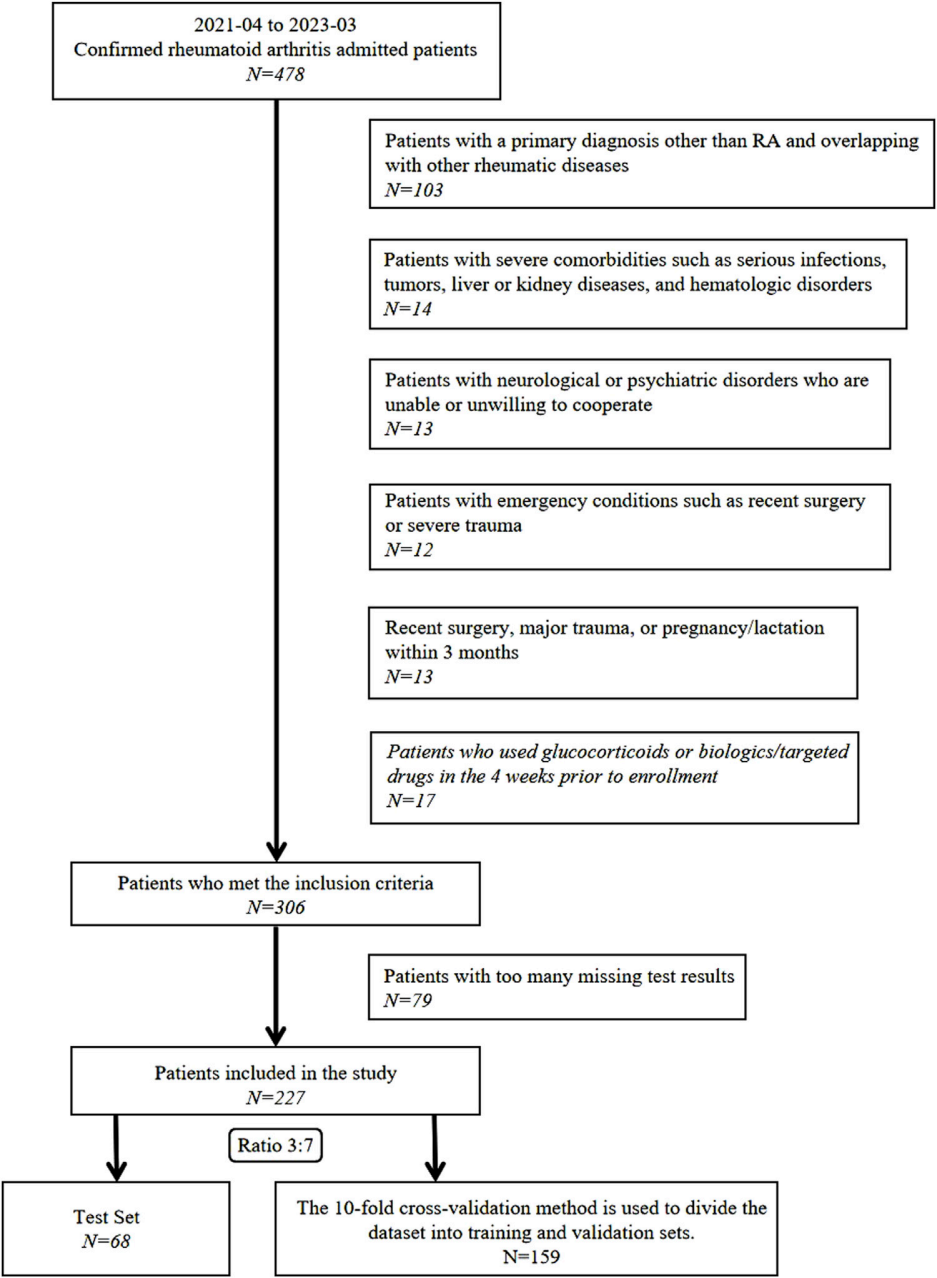
Participant screening and enrollment workflow.

This cross-sectional study was conducted in accordance with the Transparent Reporting of a Multivariable Prediction Model for Individual Prognosis or Diagnosis guidelines (Collins et al., 2015). Between April 2021 and March 2023, 478 patients diagnosed with RA and treated at the Department of Traditional Chinese Medicine Rheumatology, China-Japan Friendship Hospital, were screened for eligibility. Exclusion criteria were as follows: 1) primary diagnosis other than RA or coexisting rheumatic diseases such as systemic lupus erythematosus or Sjögren’s syndrome; 2) severe infections, malignant tumors, hepatic or renal failure, or hematologic disorders; 3) neurological or psychiatric disorders impairing protocol compliance; 4) surgical procedures, major trauma, pregnancy, or lactation within the past 3 months; 5) treatment with glucocorticoids, biologics, or targeted synthetic disease-modifying antirheumatic drugs within 4 weeks before enrollment; or 6) incomplete clinical data (Figure 1).

After applying these rigorous exclusion criteria, 251 participants were excluded, resulting in a final cohort of 227 patients with RA. This study adhered to the ethical principles of the Declaration of Helsinki and was approved by the China-Japan Friendship Hospital Clinical Research Ethics Committee (Approval No. 2020-133-K86). All participants provided written informed consent for the anonymized use of their data in subsequent clinical research.

### 2.2 Diagnostic criteria

Patient enrollment required meeting the 2010 ACR and European League Against Rheumatism classification criteria for RA (Aletaha et al., 2010).

### 2.3 Data collection

#### 2.3.1 Clinical data collection

Demographic and clinical characteristics were documented, including sex, age (years), disease duration (years), height (cm), weight (kg), body mass index (kg/m^2^), smoking history, history of alcohol consumption, and blood pressure (mmHg, measured after 5 min of rest). Morning venous blood samples were collected and analyzed at the clinical laboratory of China-Japan Friendship Hospital. Laboratory tests included: (1) hematology: white blood cell (WBC) count, red blood cell (RBC) count, hemoglobin (HGB), and platelet (PLT) count; (2) inflammatory markers: erythrocyte sedimentation rate (ESR), C-reactive protein (CRP); and (3) immunological parameters: rheumatoid factor (RF), anti-cyclic citrullinated peptide antibody (ACCP), immunoglobulins (IgA/IgM/IgG), and complement components C3 and C4.

Disease activity was assessed and classified using the DAS-28 with ESR (DAS28-ESR). Patients were categorized into remission/low activity (DAS28-ESR ≤3.2) or moderate-to-high activity (DAS28-ESR >3.2) groups. The DAS28-ESR was calculated using the validated formula (Wells et al., 2009): DAS28-ESR = $0.56\times \surd TJC28+0.28\times \surd SJC28+0.70\times \ln\left(ESR\right)+0.014\times GH$ , where TJC28 is the tender joint count (28 joints), SJC28 denotes the swollen joint count (28 joints), ESR is the erythrocyte sedimentation rate (mm/h), and GH is the patient global health score on a 0–100 mm visual analogue scale.

#### 2.3.2 Tongue features collection

To ensure image consistency and minimize confounding factors, acquisition followed a standardized protocol encompassing participant preparation, precise image capture, and quality-controlled processing.

##### 2.3.2.1 Participant preparation

Before imaging, patients completed specific preparations to optimize image quality: fasting from food and beverages (except plain water) for at least 6 h, rinsing the mouth with plain water to remove potential contaminants, and reporting dietary or medication intake within the previous 24 h to identify possible influences on tongue coating color and morphology.

##### 2.3.2.2 Image acquisition procedures

Images were captured using a standardized Tongue Diagnosis Imaging System under constant illumination to ensure uniform lighting conditions across all patients. A precise vertical shooting angle and the built-in positioning frame of the device ensured the tongue occupied a fixed proportion within the frame, thereby standardizing the field of view. Participants sat in a standardized posture with the tongue naturally relaxed and the tip gently touching the lower incisors, allowing full exposure of the midline dorsal and sublingual regions. The acquisition protocol required standardized imaging of both areas, and all raw images were de-identified immediately after capture to protect privacy.

##### 2.3.2.3 Image processing and quality control

After acquisition, all raw images underwent automatic exposure and color correction to standardize their appearance. The tongue region was subsequently cropped to focus on the area of interest and standardize the analytical field of view. Trained personnel conducted rigorous manual quality control to exclude images with issues such as improper tongue protrusion (e.g., incomplete exposure), excessive saliva obscuring features, or severe blurriness. Only qualified images were retained for analysis.

##### 2.3.2.4 Tongue image interpretation and feature grading

**FIGURE 2:**
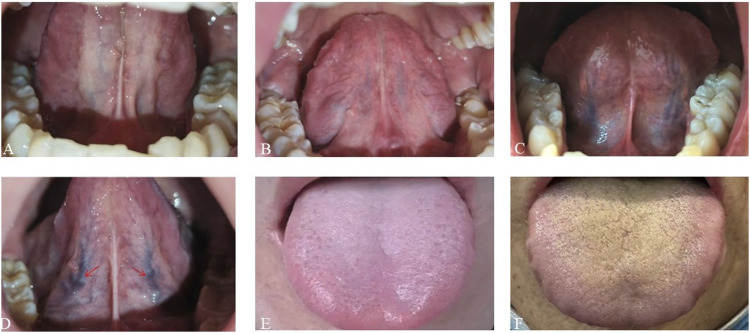
Schematic diagrams of tongue features. **(A–D)** Sublingual varicosity grading. The sublingual vein comprises submucosal veins on the ventral surface of the tongue that run alongside the lingual nerve and its tributaries. Grading is based on four parameters: vein length, diameter, tortuosity, and color. **(A)** Grade 0: Light blue or lavender veins extending ≤50% of the distance from the sublingual caruncle to the tongue tip. **(B)** Grade I Pale to bluish-purple veins extending beyond 50% of the distance without significant tortuosity. **(C)** Grade II: Dark purple veins extending beyond 50% of the distance with radial branching. **(D)** Grade III: Dark purple veins with localized nodular dilation (indicated by arrows); severe cases may show grape-like clusters. **(E,F)** Greasy coating grading. **(E)** Non-greasy coating: Filiform papillae remain discretely distributed (>50 μm inter-papillary spacing) over pink mucosa without consolidated keratinized layers. **(F)** Greasy coating: Filiform papillae merge into continuous keratinized sheets with absent interpapillary spaces and yellowish-white debris covering.

To ensure objectivity, consistency, and diagnostic reliability in interpreting tongue features, specific protocols were followed. Sublingual vein grading, alongside evaluation of tongue coating and texture, was independently performed by at least two trained traditional Chinese medicine practitioners. When discrepancies occurred, a third expert reviewer adjudicated to reach consensus. Figure 2 shows the classification criteria for sublingual vein grading.

### 2.4 Statistical analysis

Statistical analyses and visualizations were conducted using R (version 3.6.3) and Python with the scikit-learn library (version 0.22.1). Normally distributed continuous variables are expressed as mean ± standard deviation (SD), and non-normally distributed variables are presented as median (interquartile range). Categorical data are presented as frequencies and percentages. For group comparisons, the independent-samples t-test was used for normally distributed continuous variables, while the Mann–Whitney U test (Wilcoxon rank-sum test) was used for non-normally distributed variables. Categorical variables were compared using the chi-squared (χ^2^) test. For variables with <20% missing values, multiple imputation was conducted using the Multivariate Imputation by Chained Equations (MICE) package (version 3.16.0) in R to improve completeness and reduce bias from missing data.

Multivariate logistic regression (LR) analyses were performed to evaluate the predictive capacity of two models for RA disease activity. Before model development, key predictors were identified using the Boruta feature selection algorithm, which ranks variables by importance (Kursa and Rudnicki, 2010). Effect sizes for each variable were expressed as odds ratios (ORs) with 95% confidence intervals (CIs).

Receiver operating characteristic (ROC) curves were plotted using R software, and the area under the curve (AUC) was calculated to evaluate model discrimination. Differences in AUCs between models were assessed using the non-parametric DeLong test (Zou et al., 2024). Clinical utility was further examined using Decision Curve Analysis (DCA) with the rmda package (version 1.6) in R, which quantifies net benefit across clinically relevant threshold probabilities to guide risk-benefit decisions. Model calibration was evaluated using calibration curves generated in Python’s scikit-learn library (version 0.22.1) to illustrate the agreement between predicted probabilities and observed outcomes. The dataset was split via random sampling, with 30% allocated to an independent test set and 70% used for model development and validation. The latter subset underwent 10-fold cross-validation, with iterative training on nine folds and validation on the tenth. A learning curve was plotted to evaluate model performance. RA disease activity status for each patient was predicted from model-generated risk scores and compared with actual clinical outcomes. Sensitivity, specificity, positive predictive value (PPV), and negative predictive value (NPV) were calculated for both models. Net reclassification improvement (NRI) and integrated discrimination improvement (IDI) were calculated to quantify improvements in discriminative performance, reflecting improvements in correctly classifying individuals into appropriate categories (Mayaud et al., 2013; Cook and Paynter, 2011). For the multivariate logistic regression models, a nomogram was constructed to visualize the contribution of each predictor to the overall risk estimation. An online interactive web-based risk calculator was generated using an R Shiny-based web application, allowing users to enter variables and receive individualized, real-time risk predictions (RStudio Team, 2023). All statistical analyses were two-sided, with p < 0.05 indicating statistical significance.

## 3 Results

### 3.1 Analysis of cohort clinical characteristics

This study included 227 patients, with a mean DAS28 of 4.07 ± 1.57 (mean ± SD) for the entire cohort. Among them, 75 patients (33.04%) were classified as having remission or low disease activity, and 152 (66.96%) as having moderate to high disease activity. Table 1 summarizes the baseline demographic and clinical characteristics of the two groups.

**TABLE 1:** Baseline data of remission/low disease activity and moderate-to-high disease activity groups.

Variables	Total sample size (n = 227)	Remission/low activity (n = 75)	Moderate/high activity (n = 152)	*P* value^▲^
Age (years, $\bar{x}$ ± s)	58.75 ± 13.65	57.44 ± 15.63	59.39 ± 12.57	0.347
BMI(kg/m^2^)	22.47 ± 3.45	22.13 ± 3.29	22.63 ± 3.52	0.307
WBC(10^9^/L)	5.87 ± 1.82	5.20 ± 1.53	6.19 ± 1.86	0.001**
RBC(10^12^/L)	4.04 ± 0.54	4.04 ± 0.57	4.03 ± 0.53	0.951
HGB (g/L)	117.43 ± 16.92	121.47 ± 18.18	115.44 ± 15.96	0.011*
PLT (10^9^/L)	250.48 ± 85.13	223.01 ± 57.87	264.04 ± 92.98	0.001**
Female, n (%)	172 (75.8)	57 (76)	115 (75.7)	0.955
Alcohol history, n (%)	24 (10.6)	6 (8.0)	18 (11.8)	0.378
Smoking history, n (%)	27 (11.89)	6 (8.0)	21 (13.8)	0.170
Duration (years,P_25_,P_75_)	8 (3,15)	7 (2,15)	8.5 (3,16.75)	0.266
RF(IU/mL,P_25_,P_75_)	89 (23,388)	73 (21,309)	102.5 (27,438.5)	0.196
anti-CCP(U/mL,P_25_,P_75_)	546 (49,1667)	598 (78,1724)	525.5 (41.75,1637.5)	0.426
IgA(mg/dL,P_25_,P_75_)	271 (196,368)	225 (173,314)	293 (217.75,385.5)	0.001**
IgM(mg/dL,P_25_,P_75_)	117 (81,153)	117 (78,151)	118 (85.25,154)	0.66
IgG (mg/dL,P_25_,P_75_)	1,320 (1100,1630)	1,280 (1100,1520)	1,340 (1,097.5,1647.5)	0.208
Complement 3 (mg/dL,P_25_,P_75_)	88.4 (76.8,102)	82.3 (72.2,95.3)	90.4 (78.93,106.75)	0.013*
Complement 4 (mg/dL,P_25_,P_75_)	19 (15.9,23.4)	18.2 (15.5,22.6)	19.3 (15.9,24.45)	0.179

Values are presented mean ± SD., median (IQR) or percent. BMI, body mass index; WBC, white blood cell; RBC, red blood cell; HGB, hemoglobin; PLT, platelet; RF, rheumatoid factor; anti-CCP, anti-cyclic peptide containing citrulline; IgA, Immunoglobulin A; IgM, Immunoglobulin M; IgG, Immunoglobulin G; C3, complement C3; C4, complement C4.

▲ Differences between the remission/low activity and moderate-to-high activity groups were analyzed, *P < 0.05,**P < 0.01.

### 3.2 Analysis of cohort tongue features

Table 2 shows the tongue characteristics between groups. Significant group differences were observed in the distribution of tongue color, texture, coating color, coating thickness, greasy coating, and degree of sublingual varicosity (P < 0.05).

**TABLE 2:** Detailed information of cohort tongue features.

Tongue features	Total sample size (n = 227)	Grouping		Chi-square test	
		Remission/low activity (n = 75)	Moderate/high activity (n = 152)	X^2^ value	*P* value^▲^
Color of tongue body					
Red	111 (48.90%)	28 (37.33%)	83 (54.61%)	12.859	0.002**
Light red	90 (39.65%)	42 (56%)	48 (31.58%)		
Cyanotic	26 (11.45%)	5 (6.67%)	21 (13.81%)		
Shape of tongue body					
Normal	171 (75.33%)	63 (84%)	108 (71.05%)	5.09	0.078
Thin	22 (9.69%)	6 (8%)	16 (10.53%)		
Enlarged	34 (14.98%)	6 (8%)	28 (18.42%)		
Dorsal tongue mucosa					
Hyperkeratinized	101 (44.49%)	20 (26.67%)	81 (53.29%)	14.413	0.001**
Hypokeratinized	126 (55.51%)	55 (73.33%)	71 (46.71%)		
Teeth marks on tongue edges					
Absence	157 (69.16%)	58 (77.33%)	99 (65.13%)	3.506	0.061
Presence	70 (30.84%)	17 (22.67%)	53 (34.87%)		
Fissured tongue					
Absence	193 (85.02%)	64 (85.33%)	129 (84.87%)	0.009	0.926
Presence	34 (14.98%)	11 (14.67%)	23 (15.13%)		
Color of coating					
Yellow fur	88 (38.77%)	19 (25.33%)	69 (45.39%)	8.514	0.004**
White fur	139 (61.23%)	56 (74.67%)	83 (54.61%)		
Thickness of coating					
Thin fur	150 (66.08%)	60 (80%)	90 (59.21%)	9.684	0.002**
Thick fur	77 (33.92%)	15 (20%)	62 (40.79%)		
Moistness of coating					
Moist fur	199 (87.67%)	65 (86.67%)	134 (88.16%)	0.103	0.748
Dry fur	28 (12.33%)	10 (13.33%)	18 (11.84%)		
Greasy coating					
No	122 (53.74%)	56 (74.67%)	66 (43.42%)	19.722	0.001**
Yes	105 (46.26%)	19 (25.33%)	86 (56.58%)		
Coverage of coating					
Normal	188 (82.82%)	64 (85.33%)	124 (81.58%)	0.497	0.481
Below normal	39 (17.18%)	11 (14.67%)	28 (18.42%)		
Extent of sublingual varicosity					
0	98 (43.17%)	53 (70.67%)	45 (29.61%)	35.344	0.001**
Ⅰ	93 (40.97%)	19 (25.33%)	74 (48.68%)		
Ⅱ	33 (14.54%)	3 (4%)	30 (19.74%)		
Ⅲ	3 (1.32%)	0 (0%)	3 (1.97%)		

▲ Differences between the remission/low activity and moderate-to-high activity groups were analyzed, *P < 0.05,**P < 0.01.

### 3.3 Development of laboratory indicator-based model

#### 3.3.1 Prioritization of laboratory indicators

**FIGURE 3:**
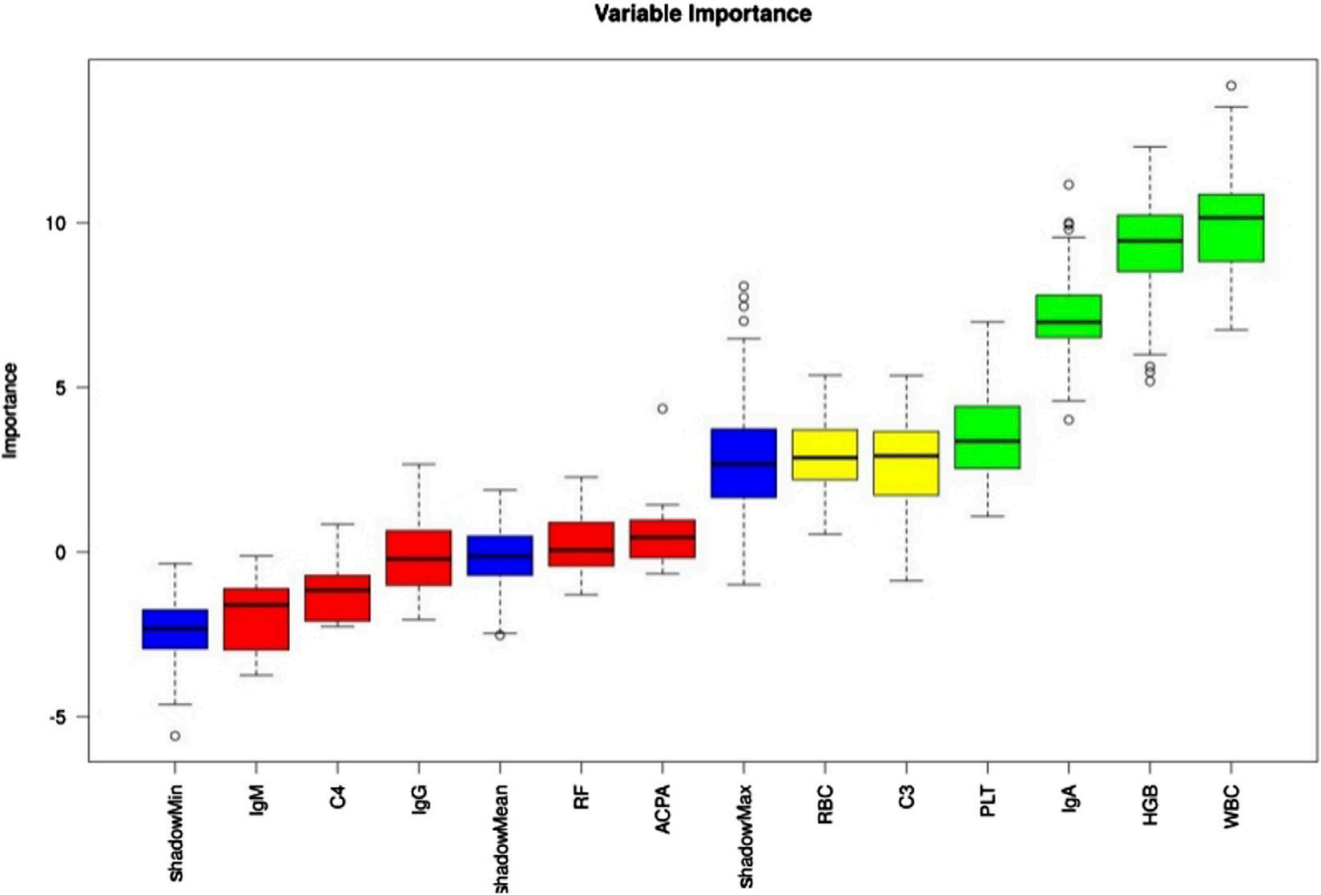
Laboratory indicator importance was assessed using the Boruta algorithm, with results color-coded as follows: green = confirmed, yellow = tentative, red = rejected.

Eleven laboratory indicators were analyzed: WBC, RBC, HGB, PLT, RF, ACCP, IgA, IgM, IgG, C3, and C4. The Boruta algorithm was used to rank these indicators based on their importance in the RA cohort, ultimately selecting four for model development in descending order: WBC, HGB, IgA, and PLT (Figure 3).

#### 3.3.2 Laboratory indicators interpretation and development of laboratory indicator-based model

A laboratory indicator-based model (Model 1) was constructed using WBC, HGB, IgA, and PLT as predictive factors. Table 3 presents their independent association with patient outcomes. WBC (OR: 1.409, 95% CI: 1.134–1.751) and IgA (OR: 1.003, 95% CI: 1.000–1.006) were risk factors for higher disease activity in patients with RA (P < 0.05 for both), while HGB (OR: 0.976, 95% CI: 0.957–0.996) was a protective factor against high disease activity (P < 0.05).

**TABLE 3:** Multivariate logistic regression analysis of laboratory indicators associated with RA Moderate-to-high disease activity.

Covariate	Regression coefficient	Standard error	Wald χ^2^	Odds ratio (95% CI)	*P*-value
WBC (10^9^/L)	0.343	0.111	9.569	1.409 (1.134,1.751)	0.002**
HGB (g/L)	−0.024	0.010	5.637	0.976 (0.957,0.996)	0.018*
PLT (10^9^/L)	0.005	0.003	3.841	1.005 (1.000,1.010)	0.050
IgA (mg/dL)	0.003	0.001	4.697	1.003 (1.000,1.006)	0.030*

**FIGURE 4:**
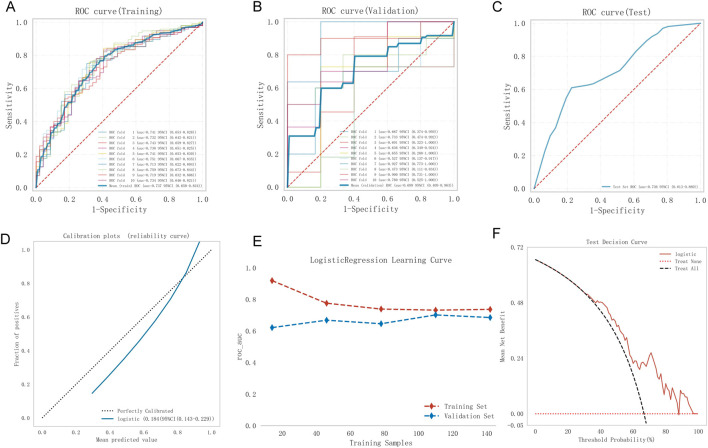
Model 1 performance. **(A)** Training ROC, **(B)** validation ROC, **(C)** test ROC, **(D)** calibration plot, **(E)** learning curves (training data: red dashed; validation data: blue dashed), **(F)** test DCA. ROC, receiver operating characteristic curve; DCA, decision curve analysis.

Model 1, developed using multivariate logistic regression, was evaluated through multiple validation metrics, including the AUC, calibration curves, learning curves, and DCA. Figures 4A–C shows that the AUC of Model 1 was 0.737 (95% CI: 0.650–0.824), 0.699 (95% CI: 0.409–0.963), and 0.736 (95% CI: 0.613–0.860) in the training, validation, and testing sets, respectively, indicating moderate discrimination across datasets. The calibration curve is closely aligned with the 45° diagonal line, suggesting good calibration (Figure 4D). As training size increased, the AUC values for the training and validation sets gradually stabilized. The convergence of AUC values indicated that Model 1 had reached its asymptotic performance. The AUC values eventually stabilized around 0.75, reflecting satisfactory discrimination and consistent generalization without overfitting or underfitting (Figure 4E). DCA further confirmed the clinical applicability of the model (Figure 4F).

### 3.4 Development of tongue feature-based model

Model 2, the tongue feature-based model, was developed by adding tongue features to Model 1. These features included tongue color and shape, dorsal mucosa, teeth marks on tongue edges, fissures, color, thickness, moistness, greasy coating, coating coverage, and extent of sublingual varicosity. After feature selection, four predictors remained: WBC, HGB, greasy coating, and extent of sublingual varicosity. Table 4 shows that WBC (OR: 1.484, 95% CI: 1.185–1.858), greasy coating (OR: 2.721, 95% CI: 1.362–5.435), and extent of sublingual varicosity (OR: 3.813, 95% CI: 2.182–6.665) were significant risk factors for high disease activity in patients with RA (P < 0.05), while HGB (OR: 0.970, 95% CI: 0.951–0.990) was a protective factor (P < 0.05).

**TABLE 4:** Multivariate logistic regression analysis of laboratory indicators and tongue features associated with RA Moderate-to-high disease activity.

Covariate	Regression coefficient	Standard error	Wald χ^2^	Odds ratio (95% CI)	*P*-value
Greasy coating	1.001	0.353	8.040	2.721 (1.362,5.435)	0.005**
Extent of sublingual varicosity	1.338	0.285	22.069	3.813 (2.182,6.665)	0.000*
WBC (10^9^/L)	0.395	0.115	11.845	1.484 (1.185,1.858)	0.001**
HGB (g/L)	−0.03	0.10	8.517	0.970 (0.951,0.990)	0.004**

**FIGURE 5:**
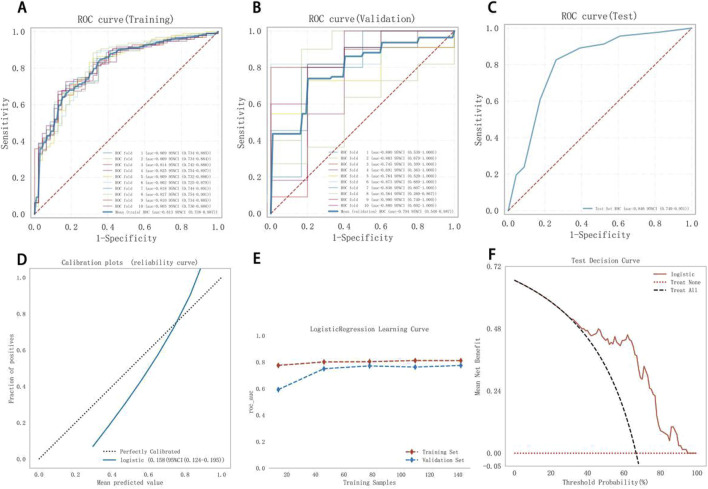
Model 2 performance. **(A)** Training ROC, **(B)** validation ROC, **(C)** test ROC, **(D)** calibration plot, **(E)** learning curves (training data: red dashed; validation data: blue dashed), **(F)** test DCA. ROC, receiver operating characteristic curve; DCA, decision curve analysis.

Figures 5A–C shows the performance of Model 2. The AUC was 0.813 (95% CI: 0.738–0.887), 0.794 (95% CI: 0.548–0.987), and 0.846 (95% CI: 0.740–0.951) for the training, validation, and test sets, respectively, indicating good discrimination. The calibration curve closely matched the ideal calibration line, with slight underestimation at probabilities >0.8 (Figure 5D). The Hosmer–Lemeshow test yielded 0.723 (>0.05), confirming a good fit. In the learning curve, the training set AUC stabilized at approximately 0.81 as the sample size increased, with no signs of overfitting (Figure 5E). DCA showed favorable clinical utility (Figure 5F).

### 3.5 Model comparison

The predictive performance of both models was systematically assessed across the training, validation, and test sets using the cutoff value, sensitivity, specificity, PPV, NPV, F1 score, and AUC (Table 5). Model 2 achieved a higher PPV than Model 1, though both models exhibited relatively low NPVs in the test set. The AUC values of Model 2 in the validation and test sets were higher than those of Model 1, indicating better discriminatory accuracy. DeLong’s test confirmed a statistically significant difference between the ROC AUCs of Models 1 and 2 (P = 0.014). According to the IDI formula: IDI = $\left(P_{new-events}-P_{old-events}\right)+\left(P_{old-nonevents}-P_{onew-nonevents}\right)$ $=\left(0.7790-0.7317\right)+\left(0.5437-0.4479\right)=0.1431$ . Since IDI >0, Model 2 demonstrated significantly improved disease activity discrimination compared to Model 1. The NRI was calculated as: $NRI=\left({\text{Sensitivity}}_{new}-{\text{Sensitivity}}_{0ld}\right)+\left({\text{Specificity}}_{new}-{\text{Specificity}}_{0ld}\right)$ $=\left(80.26\%-76.32\%\right)+\left(73.33\%-68.00\%\right)=9.27\%$ , reflecting a marked improvement in classification capacity.

**TABLE 5:** Models’ performance in predicating high disease activity of RA patients.

Model	Groups	AUC (95%CI)	Cutoff	Accuracy	Sensitivity	Specificity	PPV	NPV	F1 score
Model 1	Training set	0.737 (0.650–0.824)	0.616	0.715	0.735	0.673	0.823	0.563	0.773
	Validation set	0.699 (0.409–0.963)	0.616	0.670	0.705	0.593	0.801	0.558	0.728
	Test set	0.736 (0.613–0.860)	0.587	0.652	0.739	0.478	0.739	0.478	0.739
Model 2	Training set	0.813 (0.738–0.887)	0.644	0.755	0.742	0.780	0.877	0.611	0.800
	Validation set	0.794 (0.548–0.987)	0.644	0.704	0.705	0.687	0.833	0.560	0.753
	Test set	0.846 (0.740–0.951)	0.7	0.696	0.63	0.826	0.879	0.528	0.734

### 3.6 Model presentation and application

**FIGURE 6:**
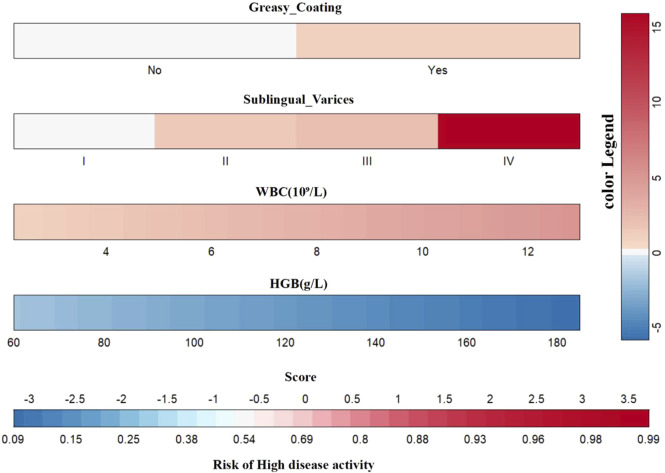
Multicolored gradient nomogram showing RA high disease activity risk scores.

To visualize the variable contributions in the modified Model 2, a color nomogram was developed (Figure 6) (Iasonos et al., 2008; Wang R. et al., 2022). The scoring system of this nomogram, derived from the multivariate logistic regression results, assigns each predictor a score proportional to its regression coefficient, indicating its effect on the risk of high RA disease activity. These scores reflect the relative influence of each predictor on the risk of high RA disease activity, with stronger effects represented by deeper colors.

**FIGURE 7:**
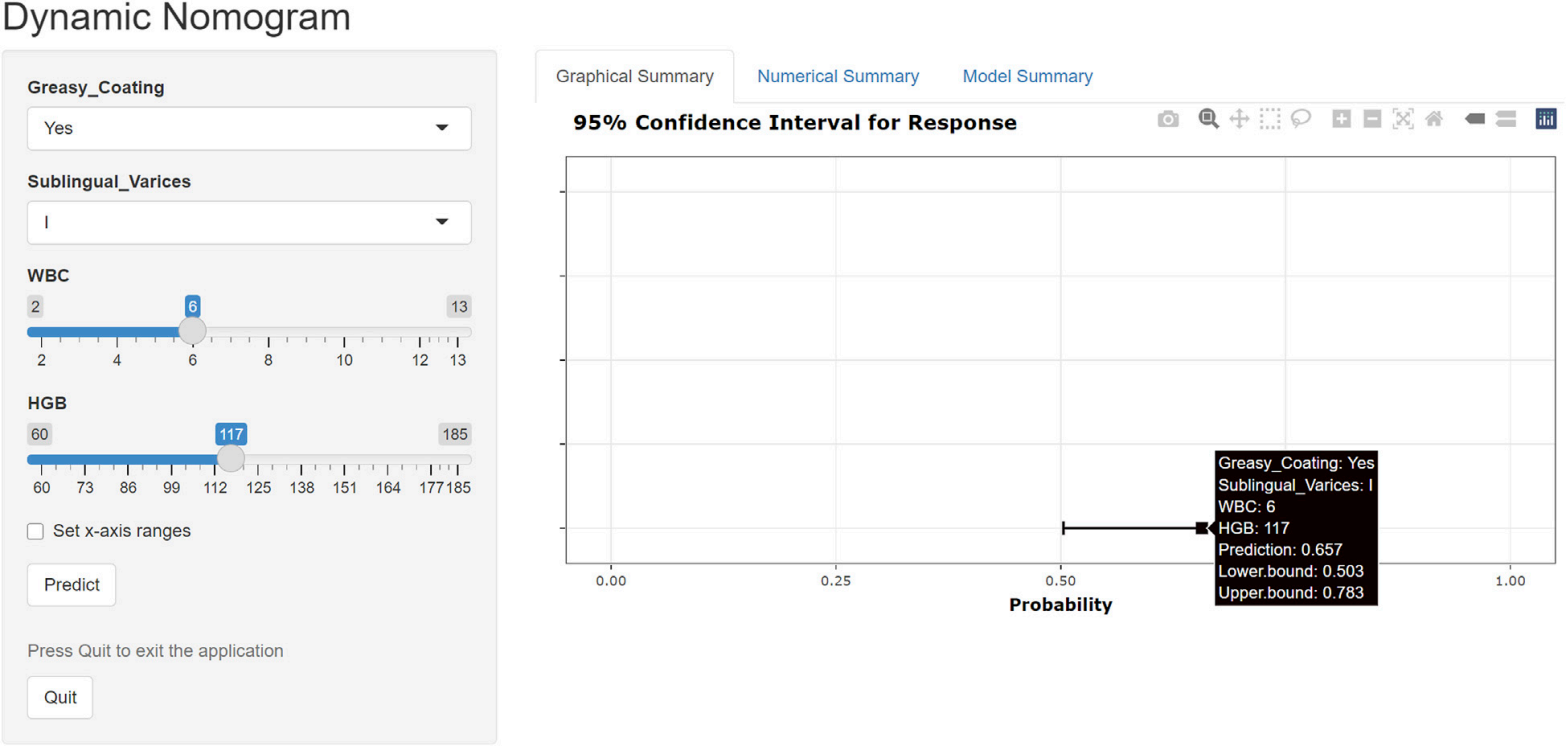
Online risk calculator created using the R Shiny platform.

An online risk calculator based on the multivariate logistic regression model was developed using the R Shiny platform (https://wangzihanprediction.shinyapps.io/WZH_Disease_activity/). By entering predictive factor values, the tool estimates the risk of high RA disease activity. For instance, for a patient with WBC = 6 × 10^9^/L, PLT = 250 × 10^9^/L, HGB = 117 g/L, greasy coating = Yes, and sublingual varicosity grade I, the calculated probability of moderate-to-high disease activity is 65.7% (Figure 7).

## 4 Discussion

Assessing RA disease activity is central to the T2T strategy. However, the ACR-recommended multidimensional evaluation system, which requires professional joint counts and complex metrics, is difficult to implement in primary care settings and for patient self-monitoring. This study integrated easily accessible tongue features, such as sublingual varicosity and greasy coating, with routine clinical indicators to develop a predictive model for RA disease activity. The integrated model (Model 2) outperformed the laboratory-only model (Model 1), suggesting that tongue features can complement traditional biomarkers and provide an auxiliary screening tool for primary care. Such a tool could help identify patients needing specialist referral, thereby optimizing referral decision-making.

Feature selection was conducted using the Boruta algorithm, a proven method for identifying robust predictors in high-dimensional datasets with multicollinearity, previously applied in cardiovascular risk assessment and oncology prognosis models (Li et al., 2025; Lin et al., 2023). Four clinical variables were identified as key contributors to the RA patient cohort model, ranked based on importance as WBC, HGB, IgA, and PLT. In patients with moderate-to-high disease activity, elevated WBC and IgA levels and reduced HGB were significant risk factors, aligning with findings from a cross-sectional study of 779 patients (Scholz et al., 2019). The underlying mechanism may involve chronic inflammation-induced hepcidin upregulation and erythropoietin inhibition, collectively lowering HGB levels. Smith et al. (Smith et al., 1992) also attribute RA-related anemia to inflammation-driven iron metabolism dysregulation and cytokine-mediated erythropoiesis suppression. Boilard et al. (2012) report that platelets, beyond mediating hemostasis, actively participate in RA-related inflammation, with activation/apoptosis dynamics closely linked to disease activity. In the final model, the ORs for PLT and IgA were near 1, and the lower bounds of their 95% CIs approached 1.000, suggesting weak statistical significance. This likely reflects the small measurement units of PLT and IgA, where significant biological effects require large cumulative changes, as well as potential attenuation of effect size from the limited sample size. However, their inclusion in the model is warranted, as Boilard et al. (2012) confirm that platelet activation drives the RA synovial inflammatory cascade by releasing pro-inflammatory mediators, correlating positively with disease activity. Elevated IgA levels are associated with specific RA subtypes (Jorgensen et al., 1992), potentially reflecting mucosal immune abnormalities that contribute to disease heterogeneity. Therefore, including PLT and IgA in the model is consistent with the algorithm screening results and the biological basis of RA inflammatory activity.

Tongue features have been recognized for over 3,000 years in China as indicators of internal health and pathophysiological states. Standard tongue features include color, dorsal mucosa, coating moistness, and sublingual varicosity extent (Wang et al., 2020). Tongue color is primarily influenced by vascularization of the lingual papillae, while the coating, composed of keratinized filiform papillae tips and exfoliated epithelial cells, is modulated by factors such as oral microbiota composition, blood-borne metabolites, and the secretory activity of mucosal and serous salivary glands (Washio et al., 2005). As a non-invasive and cost-effective diagnostic method, tongue feature analysis has recently been applied in predictive models for various diseases. For example, Li et al. developed an AI-driven deep learning diagnostic model using tongue features to differentiate patients with gastric cancer from those with non-gastric cancer, achieving superior diagnostic accuracy for early gastric cancer and precancerous lesions compared with traditional blood biomarkers (Yuan et al., 2023). Similarly, Zhuang et al. (2022) used a convolutional neural network to extract tongue features for a health assessment model, while Jiang et al. (2021) combined quantitative tongue image features, demographic data, and serological indicators in multiple machine learning algorithms to diagnose non-alcoholic fatty liver disease. Research shows that morphological and functional changes in the tongue mucosa are associated with the pathological status of various systemic conditions (Ye et al., 2014). In this study, sublingual varicosity and greasy tongue coating were independent predictors. Tongue coating thickness is regulated by the balance between epithelial cell proliferation and apoptosis, microbial community composition, and regulatory networks involving epidermal growth factor and cadherins, while tortuous dilation of sublingual veins is closely associated with vascular remodeling mediated by vascular endothelial growth factor (Li et al., 2012). These findings highlight tongue features as biological indicators of circulatory and metabolic dysregulation. Incorporating tongue features extends assessment beyond conventional biomarkers, providing intuitive and visually grounded supplementary evidence for assessing RA disease activity.

The DAS28 is a core tool in specialized clinical practice, essential for accurate disease assessment after diagnosis. However, it requires specialist-performed joint examinations and laboratory markers such as ESR or CRP, which may be difficult to obtain in primary care, rural, or resource-limited settings. In contrast, our model is convenient and of low cost, making it suitable for dynamic disease monitoring in primary care and during follow-up, thereby facilitating referrals to higher-level specialists. Future developments could include mobile applications enabling patients to capture standardized tongue images at home for upload and analysis. Combined with other indicators, this approach could support preliminary RA monitoring by primary care physicians or patients themselves. As such, this tongue image-based assessment model can effectively complement the current gold standard, DAS28. Compared with thermography-based or proteomic prediction models (Moralis-Ivorra et al., 2022; O'Neil et al., 2021), our approach delivers comparable or superior predictive performance by incorporating tongue image features while avoiding reliance on expensive equipment, thereby improving cost-effectiveness and public health applicability. However, the model demonstrated moderate NPV in the test set, suggesting caution when using it for exclusionary diagnosis. Future research should focus on further optimizing model performance by integrating additional low-cost, easily accessible multidimensional data to improve its ability to support referral decisions.

In previous research, a series of RA-specific predictive models were developed using laboratory indicators such as blood glucose, lipids, autoantibodies, and plasma metabolites to forecast clinical outcomes such as cardiovascular risk and X-ray-assessed bone destruction, all with strong clinical applicability (Wang Z. et al., 2022; Wang et al., 2025). Building on clinical indicators, this study incorporated tongue feature parameters to develop a multimodal predictive model integrating imaging omics, offering a more comprehensive approach to RA assessment. An interpretable nomogram quantified the contributions of sublingual varices and greasy coating, thereby enhancing model credibility. However, this study has limitations. First, the single-center, cross-sectional design may introduce racial or regional bias due to a geographically homogeneous patient population, limiting model generalizability. Second, as the model is still in its preliminary developmental phase, its validity has not been externally verified using independent datasets. Finally, despite applying strict exclusion criteria to control known confounders, residual interference from undiagnosed comorbidities affecting tongue features or predictive outcomes cannot be excluded. Future research should increase the sample size and incorporate multicenter, multi-regional, and multi-ethnic cohorts for external validation to strengthen model robustness and clinical applicability.

## 5 Conclusion

We developed and validated a clinical predictive model for assessing disease activity in patients with RA, using robust statistical methods that combined objective phenotypic data from standardized tongue imaging with laboratory indices. Subsequently, we implemented the model as an interactive, web-based risk calculator using the R/Shiny framework. This tool shows promise as an auxiliary resource in primary healthcare, supporting tiered diagnosis by identifying patients with RA requiring timely referral to rheumatology specialists for systematic evaluation.

## Data Availability

The original contributions presented in the study are included in the article/supplementary material, further inquiries can be directed to the corresponding author.

## Abbreviations

RA, Rheumatoid arthritis; T2T, Treat-to-target; ACR, American College of Rheumatology; DAS28, Disease Activity Score using 28-joint counts; SDAI, Simplified Disease Activity Index; CDAI, Clinical Disease Activity Index; PAS, Patient Activity Scale; RAPID-3, Routine Assessment of Patient Index Data 3; TJC, Tender joint count; SJC, Swollen joint count; WBC, White blood cell; HGB, Hemoglobin; PLT, Platelets; ESR, Erythrocyte sedimentation rate; RF, Rheumatoid factor; ACCP, Anti-cyclic citrullinated peptide antibody; MICE, Multivariate Imputation by Chained Equations; SD, Standard deviation; CIs, Confidence intervals; ROC, Receiver operating characteristic; AUC, Area under the curve; DCA, Decision Curve Analysis; PPV, Positive predictive value; NPV, Negative predictive value; NRI, Net reclassification improvement; IDI, Integrated discrimination improvement.
